# Field Pea Can Be Included in Fattening Concentrate without Deleterious Effects on the Digestibility and Performance of Lambs

**DOI:** 10.3390/ani10020243

**Published:** 2020-02-04

**Authors:** Sandra Lobón, Margalida Joy, Isabel Casasús, Pablo Jose Rufino-Moya, Mireia Blanco

**Affiliations:** Centro de Investigación y Tecnología Agroalimentaria de Aragón (CITA), Instituto Agroalimentario de Aragón-IA2 (CITA-Universidad de Zaragoza), Avda. Montañana 930, 50059 Zaragoza, Spain; slobon@cita-aragon.es (S.L.); mjoy@cita-aragon.es (M.J.); icasasus@cita-aragon.es (I.C.); pablo.rufino.moya@gmail.com (P.J.R.-M.)

**Keywords:** nitrogen balance, *Pisum sativum*, weight gain, carcass quality, plasma metabolites

## Abstract

**Simple Summary:**

The partial substitution of soybean by field pea (*Pisum sativum*), a local source of protein, has been encouraged to reduce the dependency of Europe on soybean imports. However, only up to 15% of field pea inclusion in the fattening concentrate of light lambs is recommended. The aim of this study was to evaluate the outcomes of the inclusion of field pea in the fattening concentrate on the apparent digestibility, nitrogen balance, animal performance and carcass characteristics of lambs. For that, four isoenergetic and isoproteic concentrates with different proportions of field pea (0%, 10%, 20% and 30%) were compared. The main results were that the in vivo apparent digestibility of the nutrients and retained nitrogen were not affected by field pea inclusion. The performance traits were similar among concentrates, but carcass weight and dressing percentage changed cubically with the inclusion of field pea. Therefore, inclusion of field pea in fattening concentrates above current recommendations could be advisable depending on the prices and availability of the feedstuffs.

**Abstract:**

The inclusion of different proportions of field pea (0%, 10%, 20% and 30%) for partially replacing soybean in the fattening concentrate of lambs was studied for its impact on apparent digestibility and performance during fattening. In the in vivo digestibility trial, 12 lambs (33 kg body weight) were placed in metabolic crates for two periods and received restricted amounts of concentrate and straw. The performance trial involved 54 lambs (13.4 kg body weight) that received concentrate plus straw ad libitum from weaning to slaughter. The intake of crude protein was higher in the 0% pea group than in the other groups (*p <* 0.05). The inclusion of field pea did not affect the digestibility, N retained or blood metabolites. In the performance trial, most traits were not affected, although a cubic effect of field pea inclusion on hot carcass weight and dressing percentage was observed (*p <* 0.05). The inclusion of field pea did not affect total protein, urea or β-hydroxybutyrate concentrations but it affected creatinine and cholesterol concentrations (*p <* 0.05). In conclusion, field pea can constitute up to 30% of the fattening concentrate of lambs without deleterious effects on the digestibility and performance during fattening, and with minor effects on carcass characteristics.

## 1. Introduction

In recent years, the production of grain legumes has been encouraged in the European Union (EU) and the USA. The EU promotes the use of local legumes to reduce the dependency of imported soybean. Widespread soybean cultivation has social and environmental implications (carbon footprint, land-use change, etc.) in the countries of origin [1,2] and consumption implications [3], because it is generally genetically modified, which causes its social rejection [4]. On the other hand, seed legumes are strategically important because of their suitability to different agronomic conditions [5] and their capacity to reduce greenhouse gas emissions and the use of nitrogenous fertilizers [6]. In the USA, field pea (*Pisum sativum* L.) production has increased in recent years, with low-quality pea crops being destined for ruminant feed, replacing corn [7,8].

Regarding the interest of field pea as feedstuff for ruminants, pea has a high crude protein (CP) content (25–26% of dry matter (DM)) [9] and high levels of lysine and methionine [10]. The starch content of the pea is remarkable (over 40% of DM) and is highly soluble and easily degradable [11]. However, few data are available regarding the digestibility of pea in sheep [12,13], and moreover, only up to 15% of field pea inclusion is recommended in fattening lambs [14]. There are several studies on the effect of the inclusion of pea in the diet on the performance of lambs; however, they are not conclusive, as there are reported positive effects [15,16], no effects [17,18], and negative effects [19]. The abovementioned studies differed in the rate of inclusion of pea (from 15% to 86%), the duration of the fattening period (from 42 to 98 d), lamb breed, and the initial weight of the lambs (from 10 to 34 kg), which explains the variability of the results. Therefore, the aims of this study were to evaluate the effect of increasing amounts of field pea (0%, 10%, 20% and 30%) for partially replacing soybean in the concentrate on (i) the in vivo digestibility and nitrogen balance and (ii) the performance during fattening and the carcass characteristics in Rasa Aragonesa lambs.

## 2. Materials and Methods 

All procedures used in the experiment were carried out in accordance with the Spanish guidelines for experimental animal protection (RD 53/2013) with the approval of the Institutional Animal Care and Use Committee of the Research Centre (procedure number 2014-26).

### 2.1. Experimental Site and Diets

Two experiments were conducted in the Centro de Investigación y Tecnología Agroalimentaria (CITA) de Aragón in Zaragoza (41°3′ N, 0°47′ W, Spain). The feeding treatments were 4 concentrates for fattening lambs with different rates of pea inclusion: 0%, 10%, 20%, and 30%, reducing 0%, 22%, 42% and 55% the soybean inclusion, respectively. The concentrates were formulated to be isoenergetic and isoproteic; therefore, the ingredients varied among them and are reported in Table 1.

### 2.2. Animals, Experimental Design and Sampling

#### 2.2.1. In Vivo Digestibility Trial

Twelve male Rasa Aragonesa lambs (33 ± 2.8 kg body weight (BW), 4 ± 0.3 months of age) were used in 2 consecutive experimental periods. Each experimental period lasted for 21 d, comprising 14 d for adaptation to the concentrate and 7 d for collection. The lambs were distributed into 4 balanced groups according to the date of birth, BW at the beginning of the period and the weight gain of the previous period. Each group received a concentrate with a different percentage of pea (0%, 10%, 20% and 30%). Additionally, the treatment for period 1 was taken into account in the second period, so that no lamb repeated the same treatment. The lambs were placed in metabolic crates (120 cm × 50 cm) equipped with a feeder, water dispenser, slatted floor and mesh that allowed for collection of faeces and urine separately. Urine was collected in a deposit with 100 mL of 10% (v/v) H_2_SO_4_ to reach a final pH below 3. Freedom of movement and nose to nose contact between the lambs in adjacent crates were allowed during the entire study period.

To meet the maintenance requirements, each lamb received restricted amounts of concentrate, 700 and 750 g fresh matter (FM) d^−1^ in periods 1 and 2, respectively. Lambs also received 400 g FM d^−1^ straw in both periods. Daily at 8:00 h, the amount of feed offered, refusals, faeces and urine were recorded, and composite samples per animal and per period were obtained. Feed and faecal samples were dried in an oven at 60 °C for 48 h and then ground and sieved through a 1 mm screen (Rotary Mill, ZM200 Retsch, Haan, Germany); additionally, a small part of theses samples was sieved through a 0.2 mm screen. All samples were stored in total darkness until further analysis. Urine was stored at −20 °C until N analysis.

Blood samples were collected from the jugular vein into tubes containing heparin or ethylenediaminetetraacetic acid (EDTA) before each meal was offered to the lambs at the beginning and at the end of the experimental period. Blood samples were immediately centrifuged (3000× *g* for 15 min at 4 °C) and plasma stored at −20 °C until metabolite analyses.

#### 2.2.2. Performance Trial

The experiment used 54 male, single-lactating Rasa Aragonesa lambs. At weaning, lambs (13.4 ± 0.16 kg BW; 31 ± 0.6 days of age) were divided into 4 groups balanced by BW and age and weight gain during lactation. Each group received a concentrate ad libitum with a different percentage of pea (0%, 10%, 20% and 30%) plus straw on an ad libitum basis. Within each group, lambs were randomly distributed in 3 sub-groups and placed in adjacent pens with straw as the bedding. During the first week, concentrates were offered in gradual amounts.

Lambs were weighed weekly at 8:00 h with an electronic balance (0.1 kg precision). Weight gain during fattening was calculated by regression of the BW data through the trial period. The amount of concentrate offered was recorded daily, and the number of refusals was measured weekly by pen. The intake was calculated weekly by pen. Samples of the concentrates were collected every 15 d to determine the chemical composition. Individual blood samples were collected before slaughter and processed as described above.

When lambs reached the target slaughter weight (22–24 kg BW), they were slaughtered in the experimental abattoir of the Research Centre using standard commercial procedures according to Council Regulation (EC) No 1099/2009 of 24 September 2009 on the protection of animals at the time of killing. Animals from every experimental treatment were slaughtered each week. Lambs were stunned by a lightweight captive bolt pistol and were then exsanguinated. After slaughter, the hot carcass weight was recorded. After 24 h and at 4 °C in total darkness, the cold carcass weight was obtained. The dressing percentage was calculated as

(1) $$\frac{\mathrm{hot}\;\mathrm{carcass}\;\mathrm{weight}}{\mathrm{slaughter}\;\mathrm{weight}}\times 100$$

and the carcass shrinkage was calculated as

(2) $$\left(\frac{\mathrm{hot}\;\mathrm{carcass}\;\mathrm{weight}-\mathrm{cold}\;\mathrm{carcass}\;\mathrm{weight}}{\mathrm{hot}\;\mathrm{carcass}\;\mathrm{weight}}\right)\times 100.$$

The fatness degree was obtained following the Community Scale for Classification of Carcasses of Ovine Animals and of Light Lambs [20] and scored from 12 (4+, very high) to 1 (1−, very low) utilizing the scale of 1 (low), 2 (slight), 3 (average), and 4 (high). The kidney fat deposits were extracted and weighed, and the pH of the longissimus thoracis muscle was measured with a pH meter equipped with a Crison 507 penetrating electrode (Crison Instruments, S.A., Barcelona, Spain).

### 2.3. Chemical Analyses

The chemical composition analyses of feedstuffs were run in duplicate. The contents of DM (index no. 934.01) and ash (index no. 942.05) of the feedstuffs offered, of the refusals and of the faeces were determined according to the Association of Official Analytical Chemist (AOAC) methods [21]. The N content of the feedstuffs, faeces and urine was determined following the Dumas Procedure (index no. 968.06) using a nitrogen analyser (Model NA 2100, CE Instruments, Thermoquest SA, Barcelona, Spain) [21]. The contents of neutral detergent fiber (NDFom), acid detergent fiber (ADFom), and lignin (sa) of the feedstuffs and faeces were analysed following the sequential procedure of Van Soest et al. [22] using the Ankom 200/220 fibre analyser (Ankom Technology Corporation, Fairport, NY, USA). The NDFom was assayed with a heat-stable amylase. The lignin (sa) was analysed in the ADFom residues by solubilization of cellulose with sulfuric acid. All values were corrected for ash-free content. The ether extract (EE) was determined with an XT10 Ankom extractor (Ankom Technology Corporation, Fairport, NY, USA) following the Ankom Procedure [23]. Total starch was determined according to the AOAC method (index no. 996.11) with a total starch assay kit (Megazyme, Bray, Irland) based on the use of thermostable α-amylase and amylogucosidase [24]. The gross energy was determined with an oxygen bomb calorimeter (Model Parr 1341, Parr instrument company, Moline, IL, USA).

The total tract apparent digestibility of DM, CP, NDF and ADF was calculated as (ingested − excreted)/ingested. The N retention was calculated by the difference of N consumed and the total N excreted (faecal and urinary).

Different metabolites were analysed in lamb plasma. The concentrations of total protein, cholesterol, creatinine, β-hydroxybutyrate (BHB) (enzymatic colourimetric method), and urea (kinetic UV test) were determined with an automatic analyser (GernonStar, RAL/TRANSASIA, Dabhel, India). Protocols and reagents for total protein, creatinine, cholesterol and urea analyses were provided by the analyser manufacturer (RAL, Barcelona, Spain), and reagents for BHB were supplied by Randox Laboratories Ltd. (Crumlin Co., Antrim, UK). The mean intra- and interassay coefficients of variation for these metabolites were <4.4% and <5.8%, respectively.

### 2.4. Statistical Analyses

Statistical analyses were performed using SAS 9.4 (SAS Inst. Inc., Cary, NC, USA). Data from the digestibility trial (intake, apparent digestibility, nitrogen balance and plasma metabolites) were analysed using the MIXED model based on Kenward-Roger’s adjusted degrees-of-freedom solution to account for possible unequal number of observations. The model considered the inclusion of pea and the period as fixed effects and the lamb as the random effect. BW was used as a covariate for intake and apparent digestibility analyses.

Lamb performance parameters, metabolites at slaughter and carcass data were analysed with a general lineal model (procedure GLM) with the inclusion of pea as a fixed effect. The experimental unit was the animal in all the parameters except for the total DM intake, in which the pen was the experimental unit.

The results are presented as LSMeans, and the residual standard deviation or the standard error of the mean were obtained. Polynomial contrasts were used to determine the effect of the inclusion of pea in the concentrates. Differences were considered significant at *p <* 0.05.

## 3. Results

### 3.1. In Vivo Digestibility Trial

The lambs were offered a restricted amount of concentrate, and they left no refusals of concentrate but they had refusals of straw. The inclusion of pea did not affect the intake of concentrate and straw (*p* > 0.05). The average daily intake of concentrate was 593 (±1.2) and 614 (±1.5) g/DM in periods 1 and 2, respectively. The average daily intake of straw was 248 (±14.4) and 257 (±19.8) g/DM in periods 1 and 2, respectively. The inclusion of pea did not affect the apparent digestibility of the nutrients (*p >* 0.05), although the digestibility of the concentrate with 30% pea was numerically greater than that of the rest of the concentrates (Table 2).

The inclusion of pea affected the intake of N (*p* < 0.01; Table 3), being greatest for the concentrate with 0% pea and lowest for the concentrate with 30% pea. The inclusion of pea did not affect the urinary N excretion (*p >* 0.05), whereas it linearly reduced the faecal N excretion with increasing proportion (*p <* 0.05) (Table 3).

The lambs that received the concentrate with 0% pea presented greater faecal N excretion than their counterparts that received the concentrate with 30% pea. However, the retained N and the ratio of urinary to faecal N was similar among the concentrates (*p >* 0.05). The concentrations of plasma metabolites were not affected by the inclusion of pea either at the beginning or at the end of each period (Table 4; *p >* 0.05).

### 3.2. Performance Trial

The inclusion of pea in the concentrate did not have any effect on BW, weight gain or concentrate intake of the lambs during the fattening period (Table 5; *p >* 0.05). Regarding the concentration of plasma metabolites at slaughter, the inclusion of pea linearly increased the creatinine concentration (*p <* 0.05) and had a cubic effect on the cholesterol concentration (*p <* 0.01); the lambs that received the concentrate with 10% pea presented the greatest cholesterol concentrations. However, no differences were observed in total protein, urea and β-hydroxybutyrate plasma concentrations (*p >* 0.05).

The inclusion of pea in the concentrate cubically affected the hot carcass weight and dressing percentage (Table 6; *p <* 0.05). The carcasses of the lambs fed the concentrate with 10% pea were heavier than those that were fed the concentrate with 20% pea (*p* < 0.05). The carcasses of the lambs fed the concentrate with 10% pea had a greater dressing percentage than those fed the concentrates with 0% and 20% pea (*p* < 0.05). The lambs fed the concentrate with 30% pea presented intermediate values for hot carcass weight and dressing percentage.

## 4. Discussion

### 4.1. In Vivo Digestibility Trial

The lack of differences observed among concentrates with different inclusion of soybean meal and pea agrees with the results of Zagorakis, Liamadis, Milis, Dotas and Dotas [13], who found similar nutrient digestibility coefficients with an inclusion of 14.7% pea seeds vs. soybean meal in a basal diet of alfalfa hay and ground corn grain. Conversely, the partial or total substitution of soybean by pea (24.5% and 62.8%) increased the digestibility of dry and organic matter [12]. An increased degradability of the concentrates with pea is ascribed to the greater availability of digestible N of the pea [12,25], which would enhance microbial proliferation and increase the fermentation rate in the rumen. The discrepancy in the results between the studies of Purroy, Surra, Muñoz and Morago [12] and the current experiment could be related to differences in the following: i) the substrate evaluated, i.e., the diet vs. the concentrate; ii) the forage-to-concentrate ratio in the diet, which was 30:70 in the current experiment, whereas forage intake was not mentioned in the above-cited study; and iii) other ingredients among concentrates in the current experiment.

The N intake reflected the mild differences in the CP of the concentrates because the concentrate intake was restricted. In line with the N intake, the N faecal excretion also differed, although in this case only, it was significant between the concentrates with 0% and 30% pea, without affecting N retention and N in the urine. According to Zhao et al. [26], when the N intake increased by 1 g, the N output in faeces and urine increased by 0.12 and 0.45 g, respectively, in sheep fed fresh perennial ryegrass (*Lolium perenne*). Therefore, the 1 g difference of N intake between the 0% and 30% pea treatments should have been reflected in both the faeces and urine; however, it was only reflected in the former. The different diets fed in the current experiment could have affected the partitioning of N between faeces and urine [26]. The lower N excreted in the faeces by lambs fed the concentrate with 30% pea reduces the environmental impacts in relation to the rest of the concentrates. Regarding N retention, the lack of effect of the inclusion of pea in the concentrate contradicts the results of Purroy, Surra, Muñoz and Morago [12], who reported a decrease in N retention with an inclusion of 24.5% pea in lamb diets, although their diets differed in CP content (16% vs. 15%).

### 4.2. Performance Trial

As in the current experiment, the partial replacement of soybean by pea in the concentrate of Rasa Aragonesa lambs had no effect on weight gain and dry matter intake with a similar fattening period [12,27]. Similarly, the total replacement of soybean meal by pea had no effect on the fattening parameters in Rasa Aragonesa [12,27] and Comisana and Merinizzata Italiana lambs [17,28]. However, the effect of the inclusion of pea may be genotype-dependent, as there was a reduction in weight gain with the inclusion of 40% pea in the concentrate in Merinizzata Cavone lambs but not in Merinizzara Lecesse lambs [19]. Moreover, the effect of the inclusion of pea is also dependent on the variety of pea. Moriel et al. [29] observed an improvement in weight gain in lambs when soy meal was replaced by the Forager pea variety, but not with the Carnival variety. Pea varieties present similar chemical compositions except for the condensed tannin content [30], which could have positive or negative effects depending on the content. According to Purroy, Surra, Muñoz and Morago [12], there is a positive association when soybean and pea are combined in a concentrate because there is a coupling of the rapidly fermentable N of pea and the less digestible protein of soya, part of which would reach the duodenum directly.

The effect of the inclusion of pea on carcass weight and dressing percentage in the current experiment is unclear. Most of the studies that totally replaced soybean by pea in the fattening concentrates reported no effect on both of these parameters in light lambs [18,19,28]. Accordingly, carcass weight and dressing percentage were similar in Rasa Aragonesa light lambs that received 0%, 24.5% and 62.8% pea in the concentrate [12]. However, in a previous study with the same breed, Purroy and Surra [27] observed that the lambs fed a concentrate with 24.5% pea had heavier carcasses than those fed 62.8% pea, and those fed the concentrate with 0% pea presented intermediate values. The partial replacement of soybean by pea (inclusion of 30%) in the concentrate in lambs of the Ojinegra de Teruel breed reduced the dressing percentage [31]. Therefore, it seems that in local Spanish breeds, there is a dose-dependent effect on these parameters which has not been studied in local Italian breeds.

As in the current experiment, the inclusion of 24.5% pea in the concentrate did not affect fat deposition in the carcass [27]. However, the same inclusion amount of pea increased the fatness degree and perirenal fat [12]. According to the abovementioned authors, the different response was ascribed to the decreased CP/metabolizable energy ratio in the concentrate with pea compared with that of the basal concentrate. In studies with isoenergetic and isoproteic concentrates, the replacement of soybean by 25% or 40% of pea increased fat deposition in the loin [18,19].

### 4.3. Blood Metabolites

The metabolites analysed in this study are associated with protein (total protein and urea) and energy (creatinine, cholesterol and BHB) metabolism. The lack of effect on total protein and urea in the in vivo and performance trials demonstrated that the protein balance was similar, as both metabolites are related to the ingested and/or mobilized protein. These results are in line with those of Lestingi, Facciolongo, Jambrenghi, Ragni and Toteda [16] and Facciolongo, Rubino, Zarrilli, Vicenti, Ragni and Toteda [18], who reported no effect of the inclusion of up to 30% field pea during a feeding period of more than 42 days on protein and urea content in the plasma of growing lambs. In contrast, Antunović et al. [32] reported a reduction of plasmatic urea when lambs were fed 13% or 26% field pea for 30 d. This reduction was related to a lower degradability of field pea protein, leading to decreased ammonia production in the rumen and, thus, a reduced urea content in plasma.

Creatinine is the final product of the dephosphorylation of phosphocreatine to creatine in muscle and is related to the proportion of muscle mass in lambs [33]. In the current experiment, the inclusion of pea did not affect plasma creatinine in the digestibility trial, but it affected plasma concentration at slaughter; the lambs fed the concentrate with 30% pea presented a greater creatinine concentration than that of their counterparts fed concentrates with 0% and 10% pea. Lambs finished on pea had lower creatinine concentrations than those fed pea plus sweet lupin or only sweet lupin [16], and the differences were ascribed to the greater rumen degradability of sweet lupin. However, Facciolongo, Rubino, Zarrilli, Vicenti, Ragni and Toteda [18] found similar creatinine concentrations in lambs fed concentrates, including 25% pea, 12% soybean meal or 25% lupin. The reason for the difference in creatinine concentration at slaughter in our experiment remains unclear.

Cholesterol, similar to creatinine, was only affected by the inclusion of pea in the performance trial but not in the in vivo digestibility trial, which could be related to the longer treatment duration in the former. Lamb age could have also influenced the cholesterol response to the diets, as Facciolongo, Rubino, Zarrilli, Vicenti, Ragni and Toteda [18] observed that the inclusion of 25% pea meal during 42 d had no effect on 90-day-old lambs. Plasma BHB is a ketone body synthesized in the liver after adipose tissue catabolism, and the lack of effect of pea would confirm that the animals were not under a short-term negative energy balance [34]. Similarly, Antunović, Klir, Šperanda, Ćavar, Mioč and Novoselec [32] reported similar BHB concentrations with the inclusion of 13% and 26% pea during 30 d in organic lambs.

## 5. Conclusions

Field pea can be included in the fattening concentrate of lambs above the recommended values. In the current study, field pea can constitute up to 30% of the concentrate of light lambs, reducing the soybean inclusion 42% without deleterious effects on the apparent digestibility, lamb performance or carcass characteristics. The inclusion of 30% pea is the most advisable rate, as it replaced more soybean and reduced the N excretion in faeces. However, the substitution should be decided based upon the feedstuff prices and availability in the market.

## Figures and Tables

**Table 1:** Ingredients and chemical composition of the feedstuffs.

Item	Concentrates				Straw
	0% Pea	10% Pea	20% Pea	30% Pea	
Ingredients, %					
Barley	27.3	23.0	15.5	11.4	-
Corn	25.7	15.0	7.5	9.2	-
Soybean meal	22.4	17.5	13.0	10.0	-
Wheat	20.0	20.0	25.0	30.0	-
Field pea	0.0	10.0	20.0	30.0	-
Wheat bran	0.0	8.5	12.8	6.1	-
Calcic carbonate	1.5	1.5	1.3	1.3	-
Sugarcane molasses	1.5	1.5	1.5	0.0	-
Palm oil	1.0	2.4	2.6	1.4	-
Salt	0.3	0.3	0.3	0.3	-
Vitamin, mineral supplements	0.3	0.3	0.3	0.3	-
Chemical composition ^1^					
Dry matter (DM) (g/kg)	851 (2.0)	849 (2.3)	848 (2.0)	848 (1.3)	853 (8.1)
Ash (g/kg DM)	53 (0.4)	53 (0.3)	51 (0.4)	54 (0.9)	67 (2.5)
Crude protein (g/kg DM)	199 (1.6)	194 (2.1)	195 (1.3)	188 (1.0)	28 (2.3)
NDFom (g/kg DM)	212 (8.6)	226 (8.9)	237 (9.3)	216 (6.3)	795 (3.3)
ADFom (g/kg DM)	44 (0.9)	54 (2.3)	62 (1.4)	60 (0.9)	479 (3.7)
Lignin (sa) (g/kg DM)	2 (0.4)	4 (0.8)	7 (0.9)	5 (0.8)	61 (0.6)
Ether extract (g/kg DM)	28 (1.4)	38 (0.2)	46 (0.1)	31 (1.2)	-
Starch (g/kg DM)	441 (15.0)	419 (14.3)	410 (13.3)	446(18.3)	-
Gross energy (MJ/kg DM)	19 (0.6)	19 (0.9)	20 (1.0)	19 (1.2)	-

^1^ mean (standard error).

**Table 2:** Effect of the inclusion of field pea in the concentrate on total tract apparent digestibility of the diets (in vivo digestibility trial).

Item	Pea in the Concentrate				RSD ^1^	Contrast (*p*-Values)		
	0%	10%	20%	30%		Linear	Quadratic	Cubic
Dry matter	0.670	0.676	0.663	0.710	0.0348	0.18	0.23	0.31
Organic matter	0.683	0.689	0.674	0.719	0.0317	0.20	0.24	0.28
Crude protein	0.678	0.698	0.685	0.729	0.0458	0.12	0.55	0.33
Neutral detergent fibre	0.440	0.472	0.429	0.503	0.0531	0.29	0.48	0.18
Acid detergent fibre	0.345	0.348	0.323	0.412	0.0668	0.32	0.28	0.43

^1^ residual standard deviation.

**Table 3:** Effect of the inclusion of pea in the concentrate on nitrogen (N) balance in lambs (in vivo digestibility trial).

Item	Pea in the Concentrate				RSD ^1^	Contrast (*p*-Values)		
	0%	10%	20%	30%		Linear	Quadratic	Cubic
Consumed N (g/d)	21.3 ^a^	20.8 ^b^	20.7 ^b^	20.1 ^c^	0.10	<0.001	0.44	0.004
Urinary N (g/d)	9.9	9.4	9.6	9.4	1.26	0.57	0.81	0.71
Faecal N (g/d)	6.5 ^a^	6.0 ^ab^	6.2 ^ab^	5.2 ^b^	0.86	0.03	0.53	0.23
Retained N (g/d)	4.9	5.4	4.9	5.5	1.13	0.65	0.94	0.46
Ratio of urinary to faecal N	1.6	1.6	1.6	1.9	0.33	0.13	0.45	0.58

^a,b,c^ Means within a row with different superscripts differ significantly (*p* < 0.05); ^1^ residual standard deviation.

**Table 4:** Effect of inclusion of field pea in the concentrate on plasma metabolites at the beginning and end of the in vivo digestibility trial.

Item	Pea in the Concentrate				RSD ^1^	Contrast (*p*-Values)		
	0%	10%	20%	30%		Linear	Quadratic	Cubic
Beginning of the trial								
Total protein (g/L)	38.3	33.8	42.0	42.0	7.35	0.17	0.47	0.15
Urea (mmol/L)	6.6	6.5	6.6	6.2	0.64	0.41	0.52	0.56
Creatinine (µmol/L)	94.9	111.5	117.5	112.8	16.95	0.12	0.20	1.00
Cholesterol (mmol/L)	1.1	1.0	1.1	1.0	0.26	0.89	0.47	0.52
β-hydroxybutyrate (mmol/L)	0.26	0.22	0.18	0.23	0.051	0.36	0.08	0.48
End of the trial								
Total protein (g/L)	38.7	40.7	42.2	40.5	6.14	0.55	0.48	0.83
Urea (mmol/L)	6.9	6.9	6.6	6.5	0.65	0.31	0.88	0.72
Creatinine (µmol/L)	118.2	118.6	113.9	122.0	22.33	0.87	0.69	0.68
Cholesterol (mmol/L)	1.1	1.1	1.3	1.1	0.18	0.59	0.11	0.07
β-hydroxybutyrate (mmol/L)	0.19	0.21	0.23	0.21	0.040	0.54	0.33	0.70

^1^ residual standard deviation.

**Table 5:** Effect of the inclusion of pea in the concentrate on the performance during the fattening period and on the plasma metabolites of lambs at slaughter (performance trial).

Item	Pea in the Concentrate				SEM ^2^	Contrast (*p*-Values)		
	0%	10%	20%	30%		Linear	Quadratic	Cubic
Initial BW (kg)	13.4	13.4	13.4	13.3	0.16	0.90	0.84	0.97
Final BW (kg)	23.2	23.2	23.0	23.1	0.11	0.68	0.95	0.54
Average daily gain (g/d)	240	252	247	248	6.5	0.74	0.68	0.68
Fattening period length (d)	43	40	42	42	1.3	0.95	0.63	0.76
Total DM intake ^1^ (kg)	24.4	23.4	23.5	25.7	0.96	0.66	0.43	0.90
Plasma metabolites								
Total protein (g/L)	29.6	31.2	26.1	28.3	0.95	0.29	0.88	0.10
Urea (mmol/L)	4.27	4.68	4.65	4.65	0.14	0.38	0.48	0.72
Creatinine (µmol/L)	64.1 ^b^	62.0 ^b^	75.4 ^ab^	79.8 ^a^	2.69	0.02	0.55	0.32
Cholesterol (mmol/L)	0.58 ^b^	0.77 ^a^	0.59 ^b^	0.59 ^b^	0.02	0.49	0.06	0.01
β-hydroxybutyrate (mmol/L)	0.26	0.20	0.34	0.18	0.10	0.87	0.67	0.39

^a,b,c^ Means within a row with different superscripts differ significantly (*p* < 0.05); ^1^ estimated on a pen-basis; ^2^ standard error of the mean.

**Table 6:** Effect of the inclusion of pea in the concentrate on carcass characteristics and pH of the longissimus thoracis muscle (performance trial).

Item	Pea in the Concentrate				SEM ^1^	Contrast (*p*-Values)		
	0%	10%	20%	30%		Linear	Quadratic	Cubic
Hot carcass weight, kg	10.54 ^ab^	10.93 ^a^	10.46 ^b^	10.63 ^ab^	0.1	0.80	0.51	0.04
Cold carcass weight, kg	10.24	10.57	10.17	10.30	0.1	0.74	0.50	0.07
Dressing percentage, %	45.5 ^b^	47.0 ^a^	45.4 ^b^	46.0 ^ab^	0.3	1.00	0.40	0.02
Carcass shrinkage, %	2.8	3.1	2.7	3.1	0.1	0.51	0.90	0.10
Fatness score (1–12 scale)	5.1	5.2	5.6	5.6	0.1	0.09	0.75	0.62
Perirenal fat weight, g	83	87	84	79	3.2	0.59	0.44	0.85
Fat depth, cm	2.07	2.16	2.08	1.75	0.1	0.43	0.47	0.96
pH	5.64	5.64	5.66	5.63	0.01	0.89	0.32	0.43

^a,b^ Within a parameter, means with different superscripts differ at *p <* 0.05; ^1^ standard error of the mean.
